# Research progress on osseointegration performance of porous structure-modified titanium alloy implants

**DOI:** 10.3389/fbioe.2026.1748736

**Published:** 2026-02-26

**Authors:** Xingda Huang, Xi Gong, Aofei Xu, Weiying Zhang, Yi Li, Yunfei Jia, Weizhou Yang, Na Yuan, Yin Yang, Dezhi Wang, Weiguo Bian, Jiantao Liu

**Affiliations:** 1 Department of Orthopaedics, The First Affiliated Hospital of Xi’an Jiaotong University, Xi’an, Shaanxi, China; 2 Department and Institute of Urology, Tongji Hospital, Tongji Medical College, Huazhong University of Science and Technology, Wuhan, China; 3 Department of Orthodontics, College of Stomatology, Xi’an Jiaotong University, Xi’an, Shaanxi, China; 4 Department of Ultrasonography, The First Affiliated Hospital of Xi’an Jiaotong University, Xi’an, Shaanxi, China; 5 Department of Orthopedics, Xi’an Central Hospital, Xi’an, Shaanxi, China; 6 Department of Anesthesiology, Honghui Hospital Jiaotong University, Xi’an, Shaanxi, China

**Keywords:** additive manufacturing, bone regeneration, osseointegration, porosity, titanium

## Abstract

Large bone defects remain a major clinical challenge and often require load-bearing implants for reconstruction. Titanium alloys are widely used for bone defect repair because of their favorable mechanical properties and biocompatibility; however, their high elastic modulus and limited bioactivity can cause stress shielding and insufficient osseointegration. Porous architectures have therefore been introduced to tailor the elastic modulus and promote bone ingrowth. This review summarizes current clinical applications and unresolved challenges of porous titanium implants, and integrates recent preclinical evidence on manufacturing routes and key design parameters. We analyze how pore topology (periodic versus stochastic architectures), pore size, porosity, strut diameter, and multiscale designs affect osseointegration. Overall, the review provides design-oriented insights and highlights prospects and challenges for future preclinical and clinical research to improve the osseointegration of porous titanium implants.

## Graphical Abstract

**Figure:**
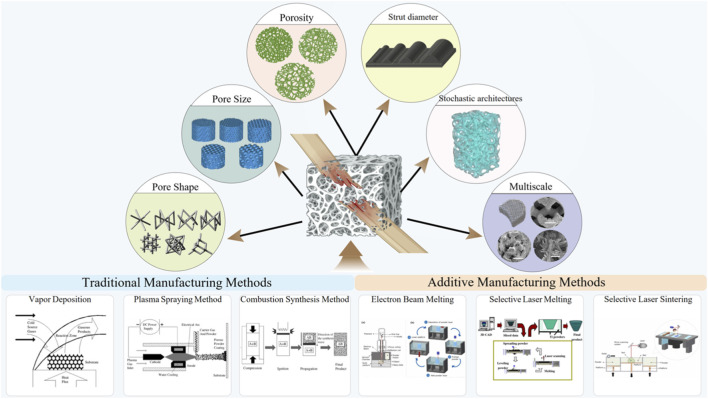
Graphical abstract summarizing key design parameters (pore shape, size, porosity, etc.) and manufacturing methods of porous titanium alloy implants.

## 1 Introduction

Large bone defects remain a major clinical challenge and often require surgical reconstruction. Once a defect exceeds a critical size, spontaneous healing is unlikely even with stabilization, and additional intervention becomes necessary. Practical criteria for critical-sized bone defects have been proposed, such as a defect length >1–2 cm together with >50% circumferential cortical bone loss, although the threshold may vary with anatomical site and local soft-tissue conditions (Nauth et al., 2018).

Implants can be manufactured from metals, bioceramics, biopolymers, or composite biomaterials (Karageorgiou and Kaplan, 2005). Metal implants are widely used because their high mechanical strength provides essential structural support and can reduce mechanical complications. Among orthopedic metal implants, titanium alloys are commonly preferred for bone defect repair because of their low density, corrosion resistance, fatigue resistance, and non-toxic, non-magnetic properties (Attarilar et al., 2021). The annual number of titanium alloy implants used in China is estimated to be as high as three million (Wang et al., 2019).

However, clinical studies have reported complications such as implant loosening and displacement, with an incidence of up to 15% in patients with osteoporosis (Liu et al., 2019; Kachooei et al., 2018). These complications are largely attributed to two limitations of Ti6Al4V: (i) the elastic modulus of Ti6Al4V (∼110 GPa) is substantially higher than that of cortical bone (17–20 GPa) and cancellous bone (∼4 GPa), which can induce stress shielding and subsequent bone resorption (Niinomi, 2008); and (ii) Ti6Al4V is relatively bioinert and lacks intrinsic bioactivity, which may compromise direct bonding with surrounding bone after implantation (Wang et al., 2023).

To address the high elastic modulus of Ti6Al4V, researchers have developed new medical titanium alloys. Significant progress has been made in the United States, Japan, and Russia with biomedical β-type titanium alloys such as Ti12Mo6Zr2Fe, Ti-Nb-Ta-Zr, and Ti51Zr18Nb (Niinomi et al., 2012; Parthasarathy et al., 2010). In China, extensive research has led to the development of alloys like Ti-24Nb-4Zr-8Sn (Ti2448) (Hein et al., 2022), Ti-10Mo-6Zr-4Sn-3Nb (Ti-B12) (Cheng et al., 2021), as well as TLE (Ti–(3–6)Zr–(2–4)Mo–(24–27)Nb) and TLM (Ti–(1.5–4.5)Zr–(0.5–5.5)Sn–(1.5–4.4)Mo–(23.5–26.5)Nb) (Zhang et al., 2007). Although these β-type titanium alloys have successfully reduced the elastic modulus to some extent, challenges remain, including complex preparation processes, high costs, and the presence of multiple constituent elements, which limit their clinical application.

Additionally, a variety of surface modification and coating strategies have been developed to enhance the biological performance of titanium alloys (Bose et al., 2018; Wa et al., 2022). These approaches aim to overcome the relative bioinertness of titanium surfaces, which can otherwise favor fibrous tissue encapsulation and ultimately contribute to implant loosening. Common surface modification methods include mechanical treatments (polishing, grinding, sandblasting), chemical treatments (acid or alkali treatment, H2O2 treatment), physical methods (thermal spraying, physical vapor deposition, ion implantation), and electrochemical methods (electropolishing, electrochemical deposition, micro-arc oxidation). Representative coatings include hydroxyapatite, calcium phosphate, polymers, and proteins (Han et al., 2023). Although these strategies can promote bone formation and vascular ingrowth and thereby improve osseointegration, they do not substantially reduce the elastic modulus and may carry risks such as coating delamination (Sheng et al., 2022). More recently, multifunctional biointerfaces for additively manufactured (AM) porous Ti/Ti6Al4V have been explored, integrating osteogenic cues with antibacterial or infection-control functions (e.g., graphene-oxide-based coatings, antibiotic-loaded hydrogels combined with MAO/PEO surfaces, and Ag/nHA-chitosan composite coatings) (Jang et al., 2024; Zhang et al., 2024; Castillejo et al., 2025).

With the continuous advancement of additive manufacturing technology, researchers have proposed using porous structures to tailor the elastic modulus and enhance the osseointegration performance of titanium alloy implants (Cheng et al., 2014; Nune et al., 2016). By adjusting the pore structure, these designs can ensure that titanium alloy implants meet the mechanical strength requirements for human use while aligning their elastic modulus with that of bone, thus significantly reducing the stress shielding effect. Moreover, porous structures provide additional space for osteoblasts, promoting their adhesion and proliferation, which facilitates the bone formation process.

Additive manufacturing (AM), commonly referred to as 3D printing, enables the fabrication of metallic implants with complex interconnected architectures and curved channels that are difficult to achieve using conventional subtractive methods (Brighenti et al., 2021). In orthopedics, AM also supports patient-specific implant fabrication based on CT/MRI reconstruction, improving geometric matching to defect sites (Smith et al., 2007; Hollister, 2005). By tuning AM process parameters, porous structures with controlled porosity can be produced to satisfy both mechanical and biological requirements. Interconnected pores facilitate vascularization and enhance the diffusion of nutrients and the removal of metabolic waste, thereby supporting cell and tissue ingrowth (Prasad et al., 2017). For example, Hou et al. reported a porous titanium scaffold with an elastic modulus of 9.7 GPa and a yield strength of 163.2 MPa, comparable to cancellous bone, and demonstrated excellent *in vitro* cell viability and osteogenic potential (Hou et al., 2022). Similarly, Deng et al. developed porous Ti6Al4V structures with elastic moduli ranging from 1.9 to 4.2 GPa, close to that of natural bone; implantation in rabbit femurs showed robust new bone formation around the implants, supporting their *in vivo* biocompatibility (Deng et al., 2021). Collectively, these findings suggest that well-designed AM porous titanium alloy scaffolds have strong potential to overcome key limitations of conventional metallic implants in load-bearing bone defect reconstruction.

The design variables of porous titanium alloy structures primarily include unit-cell topology (architecture), porosity, and pore size. These architectural parameters strongly influence the effective elastic modulus and, ultimately, the osseointegration performance of titanium alloys. Although many studies have examined how unit-cell topology, porosity, and pore size affect the mechanical properties of porous titanium alloys, a systematic synthesis of how these parameters collectively influence osseointegration remains lacking (Cheah et al., 2003a). Therefore, we reviewed and integrated the relevant literature to identify consistent patterns and provide practical guidelines for designing titanium alloy implants with optimal osseointegration, linking architectural parameters (e.g., unit-cell topology and porosity) with emerging evidence on multifunctional biointerfaces and long-term mechanical reliability.

## 2 Classification of porous scaffolds

Porous titanium scaffolds can be classified according to their architecture, which essentially reflects pore topology. In general, porous architectures can be categorized as periodic (regular) or stochastic (irregular). This classification helps reduce redundancy in subsequent discussions by separating architectural topology from geometric parameters such as pore size, porosity, and strut diameter.

### 2.1 Periodic architectures

Periodic (regular) porous scaffolds are constructed by repetitively stacking identical unit cells to form a three-dimensional architecture with a well-defined topology. With advances in computer-aided design (CAD), periodic lattices can be generated by iterative mirroring or duplication of an original 3D geometric unit (Cheah et al., 2003a; Cheah et al., 2003b). According to the unit-cell topology, periodic architectures are commonly categorized as polyhedral lattices and curved-surface-based architectures.

#### 2.1.1 Polyhedral structures

**FIGURE 1:**
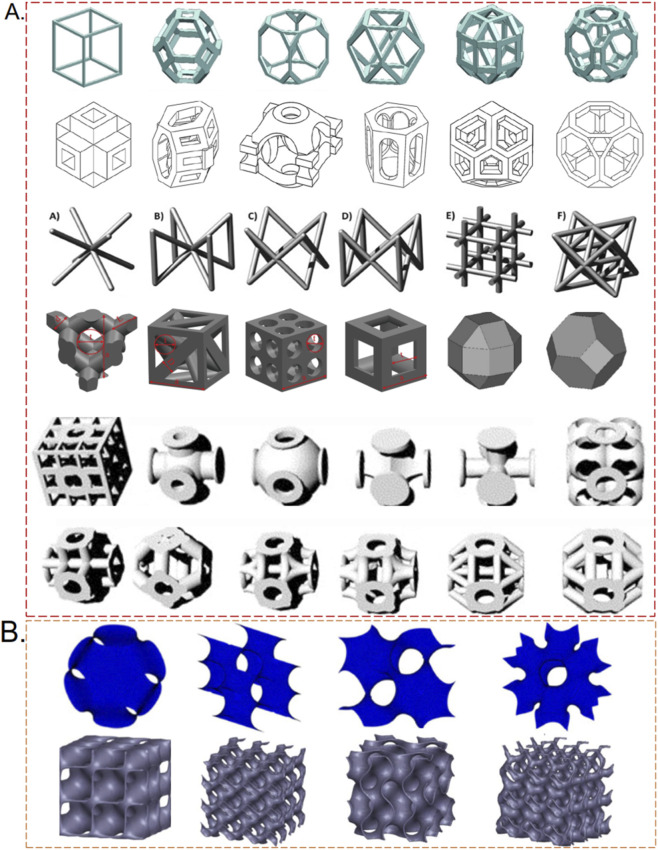
Schematic representation of porous scaffold architectures: **(A)** Polyhedral structures; **(B)** Curved porous structures.

These structures exhibit symmetry along three orthogonal axes, displaying geometric orthogonality and isotropic material properties. Polyhedral scaffolds are further divided into closed-cell and open-cell types. Closed-cell scaffolds are created by overlapping multiple polyhedral solids using Boolean operations, forming hollow structures within the gaps. In contrast, open-cell scaffolds are constructed by converting the edges of closed-cell scaffolds into struts of prescribed thickness, thereby creating an interconnected pore network (Wettergreen et al., 2005). Chantarapanich et al. proposed feasible evaluation criteria for geometric models intended for tissue scaffolds, which include: (Nauth et al., 2018): production feasibility using additive manufacturing (AM) technology; (Karageorgiou and Kaplan, 2005); polyhedral combinability, allowing single polyhedra to integrate with others; and (Attarilar et al., 2021) absence of closed pores after assembly. From 119 candidate scaffolds, three closed-cell types (truncated octahedron, rhombic dodecahedron (P-13), and rhombic cubic dodecahedron) and six open-cell types (cube, truncated octahedron, truncated hexahedron, cubic dodecahedron, rhombic cubic dodecahedron) were selected (Chantarapanich et al., 2012). Further research has led to the design of additional polyhedral scaffolds, as shown in Figure 1, which illustrates various polyhedral structures currently used as tissue scaffolds.

#### 2.1.2 Curved porous structures

The triply periodic minimal surface (TPMS) structure is a representative example of curved porous scaffolds. TPMS utilizes a fundamental unit cell characterized by smooth, continuous, non-self-intersecting periodic surfaces and crystallographic space group symmetry in three primary directions. This structure provides complex surfaces and pores, with pore shapes and size gradients that can be controlled via mathematical equations (Giannitelli et al., 2014; Feng et al., 2019; Mustafa et al., 2021). Introduced by Schwarz and Neovius between 1865 and 1883, the main TPMS structures include Schwarz P (primitive), Schwarz D (diamond), Schwarz H (hexagonal), and Neovius. In 1970, Schoen discovered the Gyroid TPMS structure (Mustafa et al., 2021). Currently, Gyroid, Diamond, and Primitive are among the most widely studied TPMS designs for porous implants because they provide highly interconnected pore networks and offer tunable mechanical and transport properties (Al-Ketan and Abu Al-Rub, 2019).

### 2.2 Stochastic architectures

From a biomimetic perspective, periodic porous structures can still differ from human trabecular bone in spatial arrangement. Trabecular bone exhibits a naturally heterogeneous (stochastic) porous architecture that can disperse stress and provide a favorable microenvironment for osteogenic cell activities (Müller et al., 1998). Therefore, trabecular-like stochastic architectures have been explored for porous titanium alloys to better emulate bone-like structural features.

Most stochastic (irregular) porous scaffolds are modeled after cancellous bone using 3D reverse modeling methods, mathematical modeling methods, or a combination of both (Mather et al., 2008; Chen et al., 2007). The reverse 3D reconstruction method designs scaffolds directly from CT and MRI data and represents a straightforward route to generating irregular porous architectures (Smith et al., 2007). Hollister et al. first proposed this approach and applied it to construct mandibular models (Hollister, 2005). In this workflow, Boolean combinations of defect and structural images derived from CT/MRI data are used to generate 3D scaffold geometries. The porosity can be coarsely tuned by adjusting rotations and overlaps (Smith et al., 2007; Hollister, 2005; Wang et al., 2016).

**FIGURE 2:**
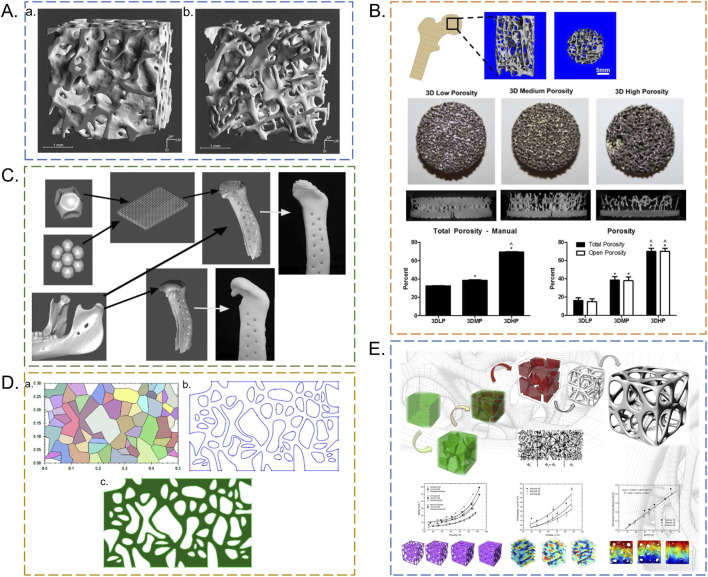
Schematic design of irregular porous scaffolds. **(A)** Three-dimensional trabecular bone structure under CT scan. **(a)** Microcomputed tomography image of trabecular bone from a 37-year-old healthy man. **(b)** Microcomputed tomography image of trabecular bone from a 73-year-old osteoporotic woman (Müller et al., 1998). **(B)** Metal scaffold of human femoral head created via reverse 3D modeling (Cheng et al., 2014). Steps include: (1) CT scanning of natural bone, (2) 3D reconstruction using Mimics software, (3) Boolean operation to generate porous structure, (4) scaffold post-processing. **(C)** Biomimetic mandible scaffold creation steps (Hollister, 2005). Key phases: (1) Defect segmentation, (2) Voronoi seed placement, (3) structural interpolation, (4) final scaffold export. **(D)** 2D Voronoi-Tessellation workflow (Kou and Tan, 2010). **(a)** Voronoi seed generation and cell partitioning, **(b)** Edge smoothing, **(c)** Pore size adjustment. **(E)** 3D Voronoi-Tessellation process (Gómez, 2016). Color mapping indicates pore size gradient: red (50–200 μm), yellow (200–400 μm), blue (400–600 μm). Merged Voronoi units simulate natural cancellous bone morphology.

Mathematical modeling methods offer ease of control and replication. The Voronoi-tessellation method is a widely used approach for designing stochastic porous scaffolds. Before its application in orthopedic implant design, Voronoi-based methods were used to model other irregular porous structures, such as tiling networks and polygonal cell-like morphologies (Nachtrab et al., 2011; Honda and Nagai, 2015). Gómez et al. first employed the Voronoi-tessellation principle to model cancellous bone, achieving agreement with key morphological parameters such as pore size and porosity (Figure 2) (Gómez, 2016).

This method involves creating Voronoi units to partition the design space into compartments, which can then be merged to generate irregular convex–concave polygons. Vertices can be modeled as control points of closed splines, and spline fitting is subsequently used to form smooth boundaries representing irregular pore shapes (Kou and Tan, 2010). This approach improves the controllability of stochastic architectures while preserving their inherent heterogeneity (Wang et al., 2018).

## 3 Methods for fabricating porous scaffolds

### 3.1 Traditional manufacturing methods

Since the discovery of the osseointegration relationship between titanium and bone tissue in 1977, titanium alloy-based implants, such as Ti6Al4V, have been widely used in orthopedic applications (Taniguchi et al., 2016). Porous titanium alloys can be categorized into non-uniform and uniform pore types, each employing distinct manufacturing methods. Traditional methods for creating non-uniform porous titanium alloys include plasma spraying, powder sintering, metal fiber sintering, the space holder method, and combustion synthesis. For uniform porous titanium alloys, commonly used methods include orderly oriented wire mesh (OOWM), ferromagnetic fiber arrays, and vapor deposition (Ryan et al., 2006). The advantages and disadvantages of these traditional manufacturing methods are summarized in Table 1.

**TABLE 1:** Common traditional manufacturing methods for titanium alloy scaffolds.

Method	Pores uniformity	Precisely controllable	Advantages	Disadvantages	References
Plasma spraying	Irregular	No	Rough surface, improves bone surface anchoring	Produces many closed pores	Hahn and Palich (1970)
Powder sintering	Irregular	No	Easy to operate	Brittle metal powder, poor toughness, material prone to cracking	Thieme et al. (2001)
Metal fiber sintering	Irregular	No	More stable mechanical structure	Difficult to manufacture complex structures	Galante and Rostoker (1973)
Space holder	Irregular	No	High porosity, nearly uniform pores	Difficult to remove a large amount of space holder material	Bram et al. (2000)
Combustion synthesis	Irregular	No	High purity of the obtained scaffold	Complicated operation	Cai et al. (2005)
Orderly oriented wire mesh	Regular	Yes	Large and uniform pore size, flexible mesh structure	Complicated operation	Ducheyne and Martens (1986)
Ferromagnetic fiber array	Regular	Yes	High porosity, regular pores	Requires ferromagnetic materials	Sivakumar et al. (1993)
Vapor deposition	Regular	Yes	Controllable porosity to a certain extent	Complicated operation	Ryan et al. (2006)

Despite their widespread use, traditional manufacturing methods present significant drawbacks, particularly the generation of substantial waste material during processing. The desired scaffold structure often remains only after extensive cutting, which can lead to imprecision and challenges in achieving accurately controlled porous titanium alloy scaffolds (Zhao et al., 2022).

### 3.2 Additive manufacturing methods

**FIGURE 3:**
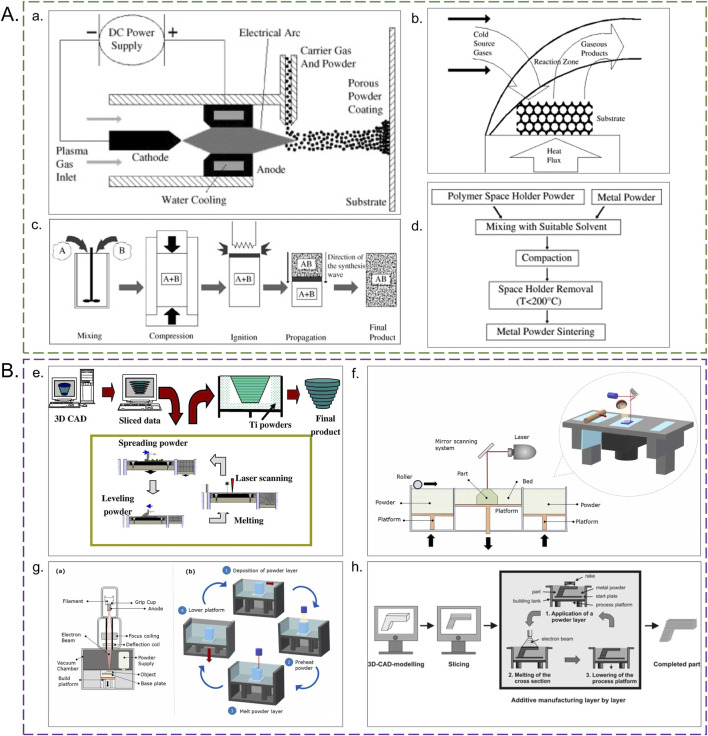
Schematic diagrams of manufacturing processes. **(A)** Traditional methods. **(a)** Plasma spraying: (1) Plasma arc generation, (2) Powder injection, (3) Porous coating formation (Hahn and Palich, 1970). **(b)** Vapor Deposition: Layered deposition of titanium vapor on a sacrificial template (Adell et al., 1970). **(c)** Combustion Synthesis Method: (1) Powder mixing, (2) Ignition, (3) Self-propagating reaction (Dabrowski et al., 2010). **(d)** Space Holder Method: (1) Polymer spacer blending, (2) Sintering, (3) Spacer removal (Dabrowski et al., 2010). **(B)** Additive Manufacturing Methods. **(e)** SLM: (1) CAD model slicing, (2) Titanium powder spreading, (3) Laser melting of powder layer, (4) Layer stacking (Heinl et al., 2008; Pattanayak et al., 2011). **(f)** SLS: (1) Powder bed preparation, (2) Laser sintering, (3) Unmelted powder removal. **(g)** EBM: (1) Vacuum chamber setup, (2) Preheating of titanium powder bed to 600°C–700°C, (3) Electron beam scanning path for selective melting, (4) Controlled cooling to prevent residual stress (Heinl et al., 2008). **(h)** EBM multi-layer stacking: Gradual formation of interconnected pores through layer-by-layer melting.

Additive manufacturing (AM), commonly known as 3D printing, constructs three-dimensional objects layer by layer based on computer-aided design (CAD) models (Christakopoulos et al., 2022). In this process, CAD models are converted into stereolithography (STL) files that describe the surface geometry of the 3D design (see Figure 3 for schematic process diagrams). The 3D printing software translates these STL files into stacked two-dimensional layers (Brighenti et al., 2021). Compared to traditional methods for creating porous structures, additive manufacturing enables the fabrication of complex 3D geometries with increased design flexibility, as well as reduced material waste and potentially lower manufacturing costs. These advantages have established AM as a predominant approach for producing orthopedic metal implants.

Several additive manufacturing methods are available, including syringe-based extrusion (EXT), fused deposition modeling (FDM), selective laser sintering (SLS), light-curing-based stereolithography (SLA), selective laser melting (SLM), digital light processing (DLP), two-photon polymerization (TPP), 3D binder jetting (3DP), and electron beam melting (EBM) (Zhao et al., 2022). Among these, SLS, SLM, and EBM are primarily employed in orthopedic implant manufacturing.

#### 3.2.1 Selective laser sintering (SLS)

This method utilizes a laser to heat and fuse powder particles layer by layer. The laser spot heats the upper powder layer, fusing it with the layer below, and the powder bed moves down after each scan. SLS technology reduces assembly time and increases material utilization, allowing for the rapid printing of complex parts, such as cavities and 3D meshes, without the need for expensive production equipment (Gu et al., 2012).

#### 3.2.2 Selective laser melting (SLM)

This technique employs a laser beam to completely melt metal powder. Although similar to SLS, SLM offers superior mechanical properties and precision (Warnke et al., 2009). Parts produced via SLM exhibit mechanical performance comparable to those made using traditional manufacturing techniques. Studies have demonstrated that specific porous titanium structures can be manufactured with SLM while retaining their biomechanical properties (Gao et al., 2022; Popovich, 2016).

#### 3.2.3 Electron beam melting (EBM)

EBM uses an electron beam under vacuum conditions to selectively melt powder layers, forming porous structures (Heinl et al., 2008; Heinl et al., 2007). This method can process brittle materials that SLM cannot, and it reduces cooling rates by increasing the powder bed temperature, thereby preventing solidification cracks. However, EBM is generally more complex and slower than SLM, resulting in higher production costs. Additionally, the vacuum atmosphere in EBM may prevent porosity formation if any adsorbed gases are present on the powder particle surfaces during manufacturing (Heinl et al., 2007).

Additively manufactured titanium scaffolds typically exhibit an as-built surface with adhered particles and pronounced roughness, which can markedly influence cell responses and osteogenic behavior (Bose et al., 2018; Cheng et al., 2014). Therefore, when comparing structural parameters such as pore size, porosity, or strut diameter, it is necessary to clarify whether the scaffold surface is in the as-built or post-processed to avoid confounding effects (Bose et al., 2018).

## 4 Pore size

The pore size of porous titanium alloy implants is a critical determinant of osteogenic potential in osteoblasts. *In vitro* studies have demonstrated significant differences in osteoblast growth dynamics between large and small pores. Research indicates that pore sizes ranging from 150 to 900 μm can adequately facilitate nutrient supply and waste diffusion, providing the design range for most contemporary titanium alloy scaffolds (Deb et al., 2018). Wo et al. employed additive manufacturing to fabricate Ti6Al4V scaffolds with pore sizes between 300 and 800 μm, conducting *in vitro* experiments that revealed enhanced cell adhesion and differentiation in scaffolds with 300 μm pores, while 800 μm pores were more effective at promoting cell proliferation (Wo et al., 2020). Larger pores enhance cell proliferation by permitting easier passage of oxygen and nutrients; however, their increased permeability may reduce the resistance of the cell suspension during seeding, thereby decreasing the duration for which cells remain adhered to the surface and subsequently lowering seeding efficiency (Ran et al., 2018). Conversely, smaller pores can enhance the expression of osteogenesis-related genes, thereby facilitating cell differentiation. This finding aligns with the experiments conducted by Teixeira et al., who designed titanium alloys with pore sizes of 312 μm, 130 μm, and 62 μm and compared the expression levels of osteogenesis-related genes (RUNX2, ALP, BSP, COL, OPN) over 14 days. They observed the highest gene expression in scaffolds with 62 μm pores, although the precise underlying mechanisms remain unclear (Teixeira et al., 2012).

Ciliveri et al. assessed the osteogenic capacity of cells *in vitro* using scaffolds with pore sizes of 670 μm and 740 μm, finding that scaffolds with 670 μm pores exhibited superior cell viability. A possible explanation for this observation is that smaller pores possess higher average curvature, which influences cell behavior. Given that the average curvature of the pore cross-section is defined as 2π/P, where P is the perimeter, larger pore sizes correspond to lower average curvature (Ciliveri and Bandyopadhyay, 2022). Furthermore, scaffolds with 670 μm pores demonstrated pore bridging among osteoblasts, positively affecting cell migration and proliferation. Mathematical analysis by Buenzli et al. indicated that the duration required for pore bridging in 3D-printed scaffolds increases linearly with initial pore size, suggesting that smaller pores facilitate shorter pore bridging times (Buenzli et al., 2020).

*In vivo* experiments, by comparison, incorporate a more complex array of factors, lending greater credence to the results. The success of *in vivo* implants hinges on various parameters, including the quantity and maturity of regenerated bone, the composition of that bone, and the strength of the bone-implant interface. Zhang et al. designed scaffolds with pore sizes of 300 μm, 600 μm, and 900 μm and conducted *in vivo* experiments in rabbits, finding that after 4 weeks, scaffolds with 600 μm pores yielded the highest levels of regenerated bone, characterized by increased trabecular formation around and within the scaffold, indicative of enhanced bone maturity (Zhang et al., 2022). Similarly, Ran et al. reached comparable conclusions, comparing scaffolds with pore sizes of 400 μm, 600 μm, and 800 μm in in vivo experiments in rats over 4 and 12 weeks, revealing that the 600 μm and 800 μm scaffolds exhibited significantly more regenerated bone than the 400 μm scaffolds (Ran et al., 2018). Larger pores may facilitate vascularization, a crucial component of *in vivo* osteogenesis, as it is generally accepted (based on *in vitro* studies) that only chondrocytes can survive beyond 25–100 μm from a blood supply (Melchels et al., 2010). Well-vascularized larger pores can directly enhance osteogenesis without necessitating prior cartilage formation, thereby significantly accelerating the bone formation process (Karageorgiou and Kaplan, 2005). However, the thickness of trabecular structures exhibited an inverse trend, with 400 μm scaffolds yielding the thickest trabecular formations, followed by 600 μm and 800 μm scaffolds. After 4 weeks, all groups displayed higher interface adhesion strength between implants and regenerated bone compared to the control group, with the 600 μm scaffold exhibiting significantly superior adhesion strength.

*In vivo* experiments in rabbits by Ciliveri, Fukuda, and Taniguchi further support the notion that pore sizes between 500 and 700 μm are favorable for titanium alloy implants in facilitating bone tissue regeneration (Taniguchi et al., 2016; Ciliveri and Bandyopadhyay, 2022; Fukuda et al., 2011). This pore size recommendation has been corroborated in various studies, including Van der Stok’s investigation, where Ti6Al4V scaffolds with approximately 500 μm pores were implanted in rat femurs, demonstrating excellent osteogenic performance across varying porosities (68% and 88%) (Van der Stok et al., 2013). Another *in vivo* study by Wieding reported favorable repair outcomes for large bone defects in sheep metatarsals utilizing SLM-manufactured Ti6Al4V scaffolds with a consistent pore size of 700 μm (Wieding et al., 2015).

In conclusion, pore size significantly impacts nutrient and waste transport, osteogenic gene expression, pore bridging, and vascularization, all of which are critical for successful osseointegration. Pore sizes ranging from 500 to 700 μm are often considered suitable for *in vivo* osteogenesis experiments in rabbits. However, challenges persist in pore size research, as the distribution of pore size and porosity are believed to exhibit a synergistic relationship, and other factors, such as overall specimen dimensions, may also confound comparisons across pore-size studies (Wang Z. et al., 2022; Loh and Choong, 2013; Pei et al., 2020). Additionally, differences between designed and manufactured pore sizes may affect the interpretation of trends in osteogenic performance and the recommendation of specific pore size ranges.

## 5 Pore shape and architecture

Pore shape can be described at different geometric levels. At the architectural level, pore topology is determined by the underlying scaffold design (e.g., periodic lattices, TPMS, or stochastic trabecular-like architectures), whereas at the local level it can refer to the pore cross-sectional geometry and curvature features that directly influence cell behavior and tissue ingrowth. The geometry of pore shapes and unit cell types in porous implants significantly influences cellular behavior, including adhesion, proliferation, and differentiation, ultimately affecting bone tissue ingrowth. Rüdrich et al. conducted *in vitro* experiments using scaffolds with five geometric pore shapes (circular, square, rhombic, star-shaped, and triangular) to assess osteoblast colonization. Their findings indicated that rhombic and triangular pores enhanced cell adhesion, while star-shaped and square pores were less conducive to osteoblast attachment. This phenomenon is attributed to the angles of their two-dimensional geometries: acute angles (θ < 90°) promote osteoblast colonization more effectively than flat edges, obtuse angles (90° < θ < 180°), or reflex angles (θ > 180°). The underlying mechanism suggests that concave surfaces offer spatial arrangement advantages for cells, resulting in greater shear stress and denser actin-myosin fiber networks, which facilitate cell migration. Cells typically settle in corners to minimize surface energy, achieving a stable state for interaction with neighboring cells (Rüdrich et al., 2019). Consequently, smaller angles provide a favorable environment for cellular interaction, while larger angles create instability due to elevated surface energy, which may hinder growth (Abbasi et al., 2020). Bidan et al. corroborated these results, developing a curvature-driven growth model validated through *in vitro* experiments utilizing various geometric configurations of non-convex symmetric pore channels (Bidan et al., 2013).

In a separate *in vitro* study, Xu et al. compared the effects of hollow hexagonal and triangular prism structures made from porous titanium alloy via selective laser melting (SLM). Their results revealed that hexagonal prism scaffolds outperformed triangular prisms in accelerating osteoblast adhesion and differentiation, although there was no significant difference in promoting cell proliferation. *In vivo* experiments in rabbits similarly showed no substantial disparity in bone formation between the two scaffold types. This inconsistency may be attributed to the larger basal surface area of hexagonal scaffolds, which increases the adhesion area and shortens the distance between angles, thereby facilitating cell attachment and spreading (Xu et al., 2022).

**FIGURE 4:**
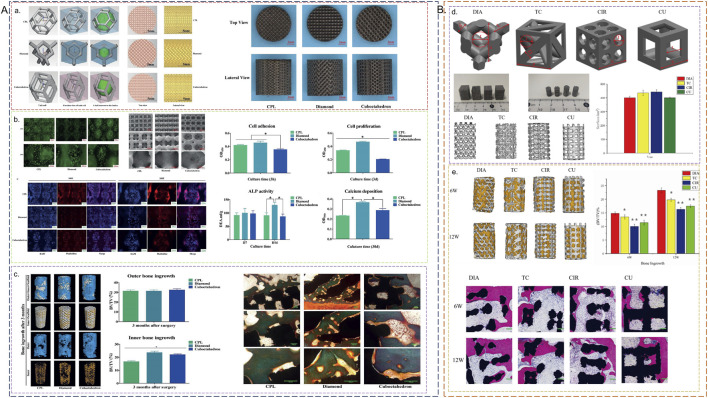
Biological properties of porous titanium alloys with different pore shapes *in vitro* and *in vivo*. **(A) (a)** Three structural elements: Cubic Pentagon Lattice (CPL), Diamond (DIA), Cubic Octahedral (COC). **(b)** Immunohistochemical staining of osteocalcin (OCN). **(c)** Micro-CT reconstruction and histological sections of porous implants with three pore shapes (Liu et al., 2023). **(B) (d)** Schematic diagram of the design parameters of the four structures (DIA, TC, CIR, CU). **(e)** Micro-CT reconstruction and histological sections of porous implants with four structures (Deng et al., 2021).

Three-dimensional (3D) porous structures introduce additional complexities compared to two-dimensional shapes, necessitating further examination of their impacts on osteoblasts *in vivo*. Deng et al. developed four distinct polyhedral structures (DIA, TC, CIR, CU) with comparable pore sizes and porosities, subsequently implanting them in rabbits and monitoring bone growth over 6 and 12 weeks (Deng et al., 2021). The diamond lattice structure exhibited superior bone growth, potentially due to its provision of increased adhesion areas for cells, minimization of internal fluid velocity differences, and longer fluid flow trajectories within the scaffold, which collectively promote vascular growth, nutrient transport, and bone formation. Huang et al. similarly found that diamond structures outperformed rhombic dodecahedrons in terms of osteogenic performance in rabbits (Huang et al., 2022). Liu et al. designed three polyhedral structures (diamond, cubic pentagonal (CPL), and cubic octahedral) and conducted both *in vivo* (rat) and *in vitro* studies, consistently demonstrating that the diamond structure exhibited the best osteogenic performance. Liu posited that the maximum shear stress observed in the diamond scaffold (120–140 MPa) may facilitate cell differentiation (Figures 4A,B) (Liu et al., 2023). Conversely, Deng et al. reported that CIR structures exhibited the least bone ingrowth, with significantly less new bone tissue formation observed in circular pores compared to square pores. This may be attributed to the tendency of circular pores to become clogged, thereby impeding nutrient and oxygen transport within the implant and negatively impacting bone ingrowth (Deng et al., 2021). In summary, concave surfaces are more conducive to osteoblast adhesion, and shorter distances between angles enhance cell attachment and spreading. Among the currently constructed 3D porous structures, the diamond structure demonstrates the most favorable biological performance, positioning it as an advantageous choice for titanium alloy implants.

### 5.1 TPMS architectures

Triply Periodic Minimal Surfaces (TPMS) are a specific category of periodic porous structures characterized by complex surfaces with nearly zero mean curvature. As with other periodic structures, TPMS can significantly affect osteogenesis by modulating the surface area, porosity, and permeability of the scaffolds. The extensive surface area of TPMS structures enhances osteoblast adhesion, while their permeability facilitates the transport of oxygen, nutrients, and waste. The pore shapes and size gradients within TPMS can be precisely controlled through mathematical modeling (Giannitelli et al., 2014). Olivares et al. contrasted TPMS scaffolds with a periodic strut-based lattice (e.g., a hexagonal unit-cell design) and found that TPMS exhibited significantly higher permeability and surface area, thereby improving cell seeding and nutrient transport. However, the uneven mechanical stimulation induced by TPMS surfaces might limit osteoblast distribution (Olivares et al., 2009). Melchels et al. demonstrated that TPMS scaffolds harbored significantly more cells than randomly porous structures after 5 days, likely due to the enhanced interconnectivity of pores that supports nutrient supply and permeability (Melchels et al., 2010). Li et al. fabricated TPMS-structured Ti6Al4V scaffolds and implanted them in pigs alongside solid scaffolds for 5 weeks. Micro-CT and Goldner trichrome staining revealed significantly greater new bone formation and more stable bone-implant interfaces in TPMS structures compared to solid scaffolds (Li et al., 2019). Importantly, recent *in vivo* and design-comparison studies have begun to address these topology-level questions, suggesting that TPMS choices (e.g., double-gyroid and topology-tailored LPBF porous Ti6Al4V) can modulate angiogenesis-related responses and bone ingrowth beyond conventional strut-based lattices (Liu et al., 2025; Kiselevskiy et al., 2024). Nevertheless, further research is essential to evaluate the osteogenic capabilities of various TPMS topologies (e.g., Gyroid, Diamond, and Primitive) relative to conventional periodic lattices, to identify the optimal TPMS design and clarify its advantages over traditional strut-based architectures.

### 5.2 Strut diameter and curvature

In the design of 3D-printed porous titanium alloy scaffolds, strut diameter is a critical micro-architectural parameter. Here, “strut” refers to the solid ligament/beam forming the unit cell of lattice scaffolds, and “strut diameter” denotes its thickness. Notably, strut diameter refers to the thickness of the lattice struts rather than the overall specimen dimensions. Together with pore size and porosity, strut diameter governs the load-bearing behavior and the available space for tissue ingrowth in lattice scaffolds, and these parameters are typically tunable by design. Pei et al. developed diamond-structured titanium alloy scaffolds with strut diameters ranging from 200 to 400 μm, which were implanted at various sites in rabbits and Beagle dogs. The results indicated that as strut diameter increased, the yield strength rose from 36.02 MPa to 180.28 MPa, and the elastic modulus increased from 1.09 GPa to 6.71 GPa. These mechanical properties align well with those of cortical bone (compressive strength: 70–150 MPa; elastic modulus: 7–30 GPa) and cancellous bone (compressive strength: 10–20 MPa; elastic modulus: 0.05–0.5 GPa). Throughout the range of strut diameters tested, the pore size (660–860 μm) and porosity (70%–90%) were maintained within commonly used ranges. Consequently, *in vivo* implantation results demonstrated good integration of the bone with surrounding tissue, with no significant differences observed between groups (Pei et al., 2020). Parthasarathy et al. employed a similar approach to fabricate porous titanium alloy structures that matched the elastic modulus of human cranial bone (Parthasarathy et al., 2011).

Among these parameters, strut diameter is closely associated with local surface curvature, which in turn regulates cell migration and osteogenic responses. Werner et al. placed MSCs on cylindrical surfaces with diameters ranging from 250 to 5,000 μm, coated with nano-scale collagen fibers oriented perpendicular to the cylinder axis. Cell migration experiments revealed that on flat or larger cylindrical surfaces (2000 μm), cells primarily migrated along the collagen fibers, a phenomenon known as “contact guidance”. However, as the cylinder diameter decreased and surface curvature increased, directional migration along the collagen fibers was disrupted. Cells increasingly aligned and migrated along the cylinder axis (zero curvature direction). Increased surface curvature was associated with elevated levels of phosphorylated myosin light chain and the preferential establishment of F-actin fibers along smaller curvature cylinders, indicating a link between curvature-induced cell bending and the F-actin-myosin mechanism that promotes longitudinal migration (Werner et al., 2018).

Pilia et al. created concentric microchannels with diameters of 100–500 μm. *In vitro* osteogenesis results demonstrated that the elastic modulus and hardness of these microchannels were significantly higher than those of flat structures, indicating that curvature significantly impacts the secretion of extracellular matrix and osteoblast mineralization. Larger curvature microchannels were found to induce faster directional osteoblast alignment and tissue formation (Pilia et al., 2013). Ciliveri et al. compared hexagonal honeycomb-like porous titanium alloy scaffolds with strut diameters of 92 μm and 116 μm. MTT (3-(4,5-dimethylthiazol-2-yl)-2,5-diphenyltetrazolium bromide) results at 3 and 7 days showed no significant differences in cell proliferation between the two sizes (Ciliveri and Bandyopadhyay, 2022). Werner et al. designed concave and convex sphere models with diameters of 250–750 μm and conducted *in vitro* experiments with MSCs. Cells on concave structures migrated significantly faster than those on convex structures and flat surfaces, while no significant difference in migration speed was observed between convex and flat surfaces. The accelerated migration on concave structures is attributed to cytoskeletal forces lifting the cell body. Conversely, cell behavior on convex surfaces was similar to that on flat surfaces, characterized by leading-edge protrusion, cell body translocation, and trailing-edge retraction. Enhanced cell differentiation on convex surfaces was due to substantial deformation of the cell nucleus induced by cytoskeletal forces, increasing lamin-A levels and promoting osteogenic differentiation (Werner et al., 2017). Park et al. found similar results, suggesting that faster cell migration on concave pores results from cells attempting to escape. They designed concave and convex microstructures with diameters of 200–300 μm and depths or heights of 50–150 μm. *In vitro* experiments indicated that cells migrated faster in concave structures but exhibited poor growth and remained round. Growth and migration on convex surfaces were similar to flat surfaces, likely due to cell membrane contact on convex surfaces leading to the release of GTPase Rac (Ras-related C3 botulinum toxin substrate), which is involved in cytoskeletal reorganization and focal complex assembly, enhancing local traction forces. Contact with concave surfaces may trigger spontaneous opening of cell membrane channels, reducing adhesion and accelerating migration (Park et al., 2009).

In summary, curvature significantly influences osteoblast behavior. Osteoblasts migrate faster on concave surfaces and exhibit similar speeds on convex and flat surfaces, while convex surfaces enhance cell differentiation. As strut diameter decreases, resulting in larger curvature, osteoblasts tend to migrate and align along the cylinder axis (zero curvature direction). The impact of curvature on cells may be related to the process of mechanotransduction (De Belly et al., 2022).

### 5.3 Stochastic architectures and heterogeneous pore distributions

Beyond periodic lattices, stochastic architectures introduce heterogeneous pore morphologies and pore-size distributions, which may better emulate trabecular bone and thereby modulate osseointegration. As previously mentioned, studies on uniform porous structures indicate that an ideal design includes a pore size between 500 and 700 μm and porosity between 60% and 90%. However, pore size influences osteogenesis differently. Research on trabecular bone shows that its natural stochastic mesh-like architecture, containing both large and small pores, provides a favorable microenvironment for osteoblast adhesion, migration, proliferation, and differentiation (Müller et al., 1998). Winther et al. implanted Zimmer Biomet porous titanium scaffolds, designed to mimic human cancellous bone, into human tibiae and followed up for 2 years. The results demonstrated good integration performance and low displacement rates (Winther et al., 2016). Therefore, researchers believe that a micro-porous structure with both large and small pores combines the advantages of different pore sizes, offering more benefits for cellular behavior than uniform-sized micro-porous structures, thus enhancing the osseointegration performance of titanium alloy implants (Wang et al., 2016; Liu et al., 2023; Dalby et al., 2007).

Reverse three-dimensional reconstruction is the most direct method for obtaining trabecular-mimicking stochastic porous scaffolds. Cheng et al. used this method to fabricate artificial titanium alloy femoral heads and conducted *in vitro* osteogenesis experiments, which showed high osteoblast activity on the scaffolds. This manufacturing method allows for rough adjustment of porosity by controlling the number of rotations and overlays. The results indicated that scaffolds with higher porosity promoted osteoblast differentiation, consistent with previous findings (Cheng et al., 2014). Pattanayak et al. implanted porous titanium scaffolds created via reverse three-dimensional reconstruction into the femoral condyles of rabbits, which also exhibited good osteogenic performance (Pattanayak et al., 2011). Bai et al. utilized computed tomography (CT) scanning to create titanium alloy acetabular cups, which were then implanted into Beagle dogs. Micro-CT and histological results showed significantly greater bone formation at 1, 3, and 6 months compared to hydroxyapatite-coated titanium alloys, indicating better performance in promoting osseointegration. However, scaffolds prepared by reverse three-dimensional reconstruction lack flexibility in custom design applications and cannot precisely control parameters such as porosity and pore size, limiting their development to some extent (Liang et al., 2019).

The Voronoi-Tessellation algorithm is a commonly used method for constructing stochastic porous structures. Unlike reverse three-dimensional reconstruction, this method allows for easier control and replication. The construction principle involves creating Voronoi cells to divide space into small partitions, which are then randomly merged to form irregular convex and concave polygons. These vertices serve as control points for closed splines, and spline curves fit the boundaries of the stochastic pores (Kou and Tan, 2010). The Voronoi algorithm enhances the controllability of simulating stochastic porous structures while maintaining the natural irregularity found in nature (Wang et al., 2018). This method can be applied to construct various models, including tiling networks and cell polygon shapes (Nachtrab et al., 2011; Honda and Nagai, 2015). Fantini et al. were the first to use the Voronoi-Tessellation technique to construct bone defect models, producing a bone model that closely resembled human cancellous bone (Fantini et al., 2016). Liang et al. employed the Voronoi algorithm to create stochastic porous Ti6Al4V scaffolds and conducted *in vitro* cell experiments, revealing that the scaffolds had good cell compatibility. Fully stochastic scaffolds (degree of irregularity = 0.5) better promoted osteoblast proliferation and differentiation. Wang et al. (Wang et al., 2021) demonstrated that biomimetic porous scaffolds with trabecular-like architectures significantly enhanced osteoblast adhesion and alkaline phosphatase (ALP) activity compared with periodic lattice scaffolds (p < 0.05), highlighting the critical role of biomimetic design in improving osseointegration. *In vitro* experiments indicated that, at constant porosity, smaller average pore sizes enhanced MC3T3-E1 cell adhesion and proliferation. *In vivo* experiments in rabbits demonstrated that scaffolds with 65% porosity and an average pore size of 550 μm exhibited the best osteogenic capacity, aligning with findings reported for periodic lattice scaffolds (Liu et al., 2023).

In addition to the Voronoi-Tessellation algorithm, other methods for designing stochastic porous scaffolds have also shown promising results. Kapat et al. utilized coagulant-assisted foaming technology to create foam-like irregular porous Ti6Al4V. Both *in vivo* and *in vitro* experiments demonstrated its excellent osteogenic properties, with scaffolds having 70% porosity and 150–200 μm pore sizes showing optimal performance (Kapat et al., 2017). Wang et al. employed Medica software to develop a stochastic porous model through Boolean operations and 3D printing. *In vitro* experiments indicated that this structure significantly enhanced osteoblast adhesion, proliferation, and differentiation (Wang et al., 2020). Furthermore, when comparing the *in vivo* osteogenic performance of these irregular scaffolds with periodic lattice scaffolds, the irregular pore scaffolds exhibited superior vascularization and new bone formation, making them more effective for bone defect repair (Wang et al., 2021).

In summary, stochastic porous structures, which combine large and small pores, better promote osteoblast adhesion, proliferation, and differentiation, facilitating new bone formation. Mathematical modeling methods like the Voronoi-Tessellation algorithm offer both controllability and stochasticity, positioning them as a key direction for future development in bone defect models.

### 5.4 Multiscale architectures

Beyond macroscopic pore-shape topology, multiscale architectures integrate macro-porosity with micro- or nano-scale features, enabling concurrent regulation of mechanical behavior and cell-instructive surface cues. As previously discussed, titanium alloy porous scaffolds offer multiple advantages in promoting bone formation. However, titanium alloys inherently lack bioactivity, and smooth surfaces without specific modifications hinder cell adhesion and proliferation (Agarwal and García, 2015; Wysocki et al., 2016). Studies have shown that implants with a neat matrix, small surface area, and zero curvature do not significantly promote osteogenesis (Gamsjäger et al., 2013). In contrast, rough surfaces with negative curvature and larger surface areas are more conducive to osteogenesis (Kommareddy et al., 2010).

Knychala et al. created an open pore slot system with varying widths and conducted *in vitro* cell experiments, revealing that rougher surfaces accelerated cell migration (Knychala et al., 2013). Similarly, Hou et al. developed a hydrogel with controllable nano- and micron-level surface roughness, using human bone marrow mesenchymal stem cells (MSCs) in in vitro experiments. Their findings indicated that higher surface roughness on hydrogels led to significantly increased cell spreading areas and more organized actin stress fibers, suggesting that rough surfaces enhance MSC migration and proliferation.

Hou et al. attributed these effects to the mechanotransduction pathway, where focal adhesion kinase (FAK), a signaling protein involved in focal adhesion formation, becomes phosphorylated under mechanical force. This phosphorylation reflects cell contractility and the activation of related signaling pathways. In MSCs attached to rough surfaces, FAK expression was significantly upregulated. Moreover, downstream RhoA/ROCK signaling aids in activating non-muscle myosin II motor proteins and forming cell stress fibers. Inhibiting ROCK protein significantly restricted cell spreading and minimized differences between surfaces with varying roughness, indicating that RhoA/ROCK signaling and myosin II contraction contribute to cell sensitivity to roughness and stiffness stimulation (Hou et al., 2020).

Consequently, several experiments suggest that hierarchical porous scaffolds with macro-, micro-, and even nano-structures may be more effective in enhancing osteogenesis compared to those with only macroscopic structures (Wa et al., 2022; Shabalovskaya et al., 2008; Zwahr et al., 2017; Kuczyńska et al., 2018). Multiscale porous scaffolds typically encompass both macroscopic and microscopic topological structures. While macroscopic structures have been discussed earlier, microscopic topological structures build upon these macroscopic frameworks (Pérez et al., 2012). Microscopic structures consist of fixed-size, periodically spaced micro-patterns, which can be produced using Direct Laser Interference Lithography (DLIL). DLIL enables the creation of periodic, controllable micro-shapes or microstructures with varying surface roughness (Kuczyńska et al., 2018; Lei et al., 2021; Deligianni et al., 2001; Ponader et al., 2008). Generally, microscopic pores range from 10 to 100 μm in size, whereas macroscopic pores are approximately 100–1,000 μm.

Research has shown that osteoblasts prefer concave micro-surfaces over convex ones for optimal growth (Gamsjäger et al., 2013; Bidan et al., 2012). Lei et al. fabricated multiscale porous scaffolds with micro-pore sizes of 20, 30, and 80 μm and conducted *in vitro* cell experiments. The results indicated that a micro-pore size of 30 μm optimized cell proliferation, closely matching the size of natural osteoblasts (MC3T3-E1). This may be because cells cannot effectively utilize their pseudopodia to cover overly large pores, resulting in distribution along steep pore walls, which hinders growth. Conversely, when pore sizes are similar to cell size, pseudopodia can latch onto the pores, enhancing cell adhesion and spreading, thereby accelerating proliferation. Lei et al. likened this process to the orderly transplantation of rice seedlings in a field, where evenly distributed seedlings have sufficient growth space, leading to healthy growth. Similarly, cells seeded onto scaffolds with periodically spaced micro-pores tend to migrate into these pores during proliferation, mirroring the distribution of rice seedlings in a field (Lei et al., 2021).

In conclusion, incorporating micro-pore structures into macroscopic scaffolds enhances osteoblast behavior. A well-designed combination of macroscopic and microscopic structures in titanium alloy scaffolds can significantly improve bone defect repair (Pei et al., 2021).

## 6 Porosity

Porosity is defined as the ratio of pore volume to the volume of the material (Lei et al., 2020). It is one of the most critical factors influencing bone formation. Total porosity (Π) can be quantified using the following formula based on the gravimetric method (Hu et al., 2002; Zhang et al., 2021).

$$\Pi =1-\left(\rho \_\text{scaffold }/\;\rho \_\text{material}\right)$$

In this equation, ρ_material represents the density of the material, while ρ_scaffold denotes the apparent density of the scaffold, calculated by dividing the scaffold’s mass by its volume (Karageorgiou and Kaplan, 2005). Common pore-size designs are summarized in Table 2.

**TABLE 2:** Common pore structure designs.

Fabrication technique	Topological structure	Pore size range (μm)	Porosity (%)	Type of study	Optimal pore size (μm)	References
EBM	Hollow circular cylinder	300, 600	70	*in vitro* and *in vivo*	300	Lu et al. (2022)
PM	Salt leaching titanium plate	154.8–531.1	40.9–53.3	*in vitro*	191.6 ± 3.7	Yao et al. (2021)
SLM^a^	Diamond molecular structure	300–800	43.3, 58.1	*in vitro*	800	Wo et al. (2020)
SLM^a^	Hexagonal honeycomb	580–740	7.5–18.8	*in vitro*	670	Ciliveri and Bandyopadhyay (2022)
SLM	Cylinder with square pores	400–900	70	*in vivo*	600–700	Zhang et al. (2022)
SLM	Diamond lattice	300–900	65	*in vivo*	600	Taniguchi et al. (2016)
SLM	Channel structure with 4 longitudinal square channels	500–1,200	—	*in vivo*	500–600	Fukuda et al. (2011)
VDB + EDM	Titanium meshes	188, 313, 390	70	*in vitro* and *in vivo*	313, 390	Chang et al. (2016)
SLM^a^	Hollow circular cylinder	500–900	—	*in vitro* and *in vivo*	600–700	Ran et al. (2018)

PM, powder metallurgy; VDB, vacuum diffusion bonding; EDM, electrical discharge machining.

^a^ All designs were generated using Computer-Aided Design (CAD) software.

When compared to solid titanium alloy implants, maintaining a certain porosity in porous titanium alloy scaffolds theoretically increases the scaffold’s surface area and enhances permeability. This promotes oxygen and nutrient transport, facilitates bone cell migration, and provides an expanded surface area for new bone formation (Cavo and Scaglione, 2016; Dziaduszewska and Zieliński, 2021). Porosity can be categorized into open and closed pores; closed pores do not contribute to functionality but are included in the total porosity calculation. In contrast, open pores enhance pore interconnectivity, facilitating cell communication, angiogenesis, and improved osseointegration, thus playing a vital role in the functional performance of the scaffold (Fousová et al., 2017). This section primarily discusses the impact of open porosity on osseointegration.

Hou et al. designed a series of titanium alloy porous scaffolds with porosities ranging from 50% to 70% and conducted *in vitro* experiments. The results indicated that cells proliferated and differentiated more effectively on scaffolds with 70% porosity, although adhesion was slightly lower compared to scaffolds with 50% and 60% porosity. This could be attributed to the higher permeability of scaffolds with greater porosity, which leads to increased fluid flow during cell seeding. *In vitro* experiments demonstrated that elevated fluid flow can reduce the time cells spend adhering to the scaffold surface, consequently diminishing adhesion. However, high permeability also facilitates oxygen, nutrient, and waste transport, creating a more favorable environment for cell proliferation. Notably, cells on scaffolds with 70% porosity exhibited the highest expression of osteogenesis-related genes such as ALP, RUNX2, and OPN, indicating enhanced osteogenic cell differentiation, although the exact mechanisms remain unclear (Fousová et al., 2017).

Zhang et al. designed porous titanium alloy scaffolds with controlled pore sizes between 600 and 700 μm and porosities of 40%, 70%, and 90%. *In vitro* experiments showed that after 7 days of culture, cell numbers on scaffolds with 70% and 90% porosity were comparable and significantly higher than those on scaffolds with 40% porosity. After 14 days, cells on scaffolds with 70% porosity exhibited significantly higher expression levels of ALP, RUNX2, and COL-1 compared to the 40% and 90% groups, while scaffolds with 90% porosity showed slightly elevated expression of OCN compared to the 70% group. Therefore, *in vitro* findings suggest that titanium alloy scaffolds with 70% porosity are favorable for promoting osteogenesis (Zhang et al., 2022).

*In vivo* studies, which account for more complex interactions than *in vitro* experiments, have similarly shown that higher porosity can enhance osteogenesis. For example, Zhang et al. reported in rabbits that a decreasing trend in bone volume ratio (BV/TV), trabecular number (Tb.N), and trabecular thickness (Tb.Th) among scaffolds with porosities of 90%, 70%, and 40%, all of which were statistically significant. This trend may be attributed to higher permeability, which ensures sufficient oxygen supply and creates more space conducive to angiogenesis. In contrast, lower porosity may lead to pore clogging, resulting in inadequate nutrient and oxygen supply, which could hinder cell growth or even induce cell death (Zhang et al., 2022). Li et al. compared two groups of porous titanium alloy scaffolds created through sintering, with porosities of 50% (average pore size 290 μm) and 75% (average pore size 460 μm). The results revealed that scaffolds with 75% porosity exhibited significantly higher rates of bone formation and bone-implant contact than those with 50% porosity (Li et al., 2018). Zheng et al. employed a salt-leaching method to fabricate titanium alloy scaffolds with porosities between 30% and 50%. After 3 months, scaffolds with 50% porosity demonstrated significantly greater bone formation compared to the other two groups in rabbit models, aligning with previous findings (Zheng et al., 2019). However, it is important to note that due to manufacturing method limitations, these experiments did not fully eliminate the influence of differing pore sizes, which represents a significant constraint.

A notable conflict exists between achieving higher biological performance and maintaining stronger mechanical properties. While increased porosity can reduce the elastic modulus, mitigate stress shielding, and enhance the compatibility of the mechanical properties of the implant with surrounding bone, it may simultaneously diminish the scaffold’s strength and stability, potentially failing to meet mechanical requirements (Arabnejad et al., 2016). Nonetheless, the mechanical properties of porous titanium alloy materials are inherently robust, leading some studies to suggest that even with very high porosity (>90%), this concern may not be prevalent (Zadpoor, 2015). Considering the findings from both *in vivo* and *in vitro* experiments, alongside the structural characteristics of titanium alloy materials, a porosity range of 60%–90% is regarded as a reasonable design standard.

## 7 Coatings and surface functionalization of 3D-Printed titanium scaffolds

Titanium alloys are intrinsically bioinert, and the biological performance of additively manufactured porous scaffolds is strongly influenced by surface chemistry and micro/nano-topography. Therefore, surface functionalization has become an important approach to enhance osseointegration and introduce additional functions (e.g., antibacterial activity) without changing the global scaffold architecture (Bose et al., 2018; Wa et al., 2022; Han et al., 2023; Sheng et al., 2022; Wang R. et al., 2022).

Micro-arc oxidation (MAO)/plasma electrolytic oxidation (PEO) is particularly attractive for 3D-printed titanium scaffolds because it can generate a firmly bonded porous oxide layer and enable incorporation of bioactive species (e.g., Ca/P) from the electrolyte, thereby improving surface wettability and osteogenic responses (Han et al., 2023; Sheng et al., 2022; Wang R. et al., 2022). Notably, MAO/PEO can be applied after printing and is compatible with complex porous geometries, although achieving uniform modification inside deep interconnected pores may remain challenging (Sheng et al., 2022; Wang R. et al., 2022).

Beyond MAO/PEO, surface modification of AM titanium scaffolds can also be achieved via mechanical or chemical post-treatments and coating-based strategies, providing a broader toolbox for improving bioactivity without altering the bulk architecture (Han et al., 2023; Sheng et al., 2022). For example, a hierarchical biofunctionalized 3D-printed porous Ti6Al4V scaffold was reported to enhance osteoporotic osseointegration through osteoimmunomodulation (Wa et al., 2022).

## 8 Frontier topics in titanium alloy scaffolds

### 8.1 Smart coatings

Smart coatings are increasingly explored to endow 3D-printed titanium scaffolds with multifunctionality beyond mechanical support, particularly antibacterial activity and enhanced osteogenic responses, while preserving the porous architecture (Bose et al., 2018; Sheng et al., 2022; Wang R. et al., 2022). Typical approaches include ion-doped or composite coatings and therapeutic layers designed for local delivery to mitigate infection and promote bone regeneration (Bose et al., 2018; Sheng et al., 2022; Wang R. et al., 2022). Representative studies have further demonstrated hierarchical biofunctionalization strategies to improve osteogenic performance under compromised bone conditions (Wa et al., 2022).

### 8.2 4D printing technologies

4D printing extends conventional 3D printing by enabling time-dependent, stimulus-responsive changes in shape or material properties through the integration of additive manufacturing, smart materials, and programmed structural design (Agarwal et al., 2021). In biomedical engineering, 4D-printed constructs have been investigated for adaptive fitting, minimally invasive deployment, and on-demand functions such as controlled drug delivery, with most studies focusing on shape-memory polymers and stimuli-responsive hydrogels (Agarwal et al., 2021; Arif et al., 2022). Although current 4D implementations are predominantly polymeric, the concept remains relevant to titanium alloy scaffolds through hybrid designs that combine load-bearing additively manufactured titanium lattices with responsive components or surface functional layers; however, key barriers to translation include reliable actuation under physiological conditions, long-term mechanical stability and fatigue resistance, and robust process validation for multi-material systems (Arif et al., 2022; Ahmed et al., 2021).

### 8.3 Functionalized surfaces for enhanced osseointegration

Surface functionalization remains a key route to improve the osseointegration of 3D-printed titanium scaffolds, because cellular responses are highly sensitive to surface chemistry and micro/nano-topography (Han et al., 2023; Sheng et al., 2022). A major unresolved challenge is to achieve uniform and durable functionalization throughout deep interconnected pores, together with verifiable process–structure–function validation under physiologically relevant conditions, to enable meaningful cross-study comparisons (Sheng et al., 2022). Future efforts are expected to move toward multifunctional surfaces that couple osteogenic and antibacterial performance while establishing standardized characterization and *in vivo* evaluation protocols to reduce confounding effects when comparing scaffold designs (Sheng et al., 2022; Wang R. et al., 2022).

## 9 Conclusion

Additively manufactured porous titanium alloy scaffolds exhibit favorable mechanical performance, low cytotoxicity, and good biocompatibility, supporting their potential as an alternative to conventional orthopedic implants. Their designability enables complex three-dimensional architectures, improving structural versatility while reducing material waste and potentially lowering manufacturing costs.

Evidence from preclinical studies indicates that key structural parameters—including pore shape, pore size, porosity, and strut diameter—affect osseointegration outcomes in animal models (e.g., rabbit femoral defect models). In general, larger strut diameters increase mechanical strength, whereas surface curvature and the associated local mechanical cues can influence cell adhesion and proliferation.

Stochastic, trabecular-mimicking porous architectures may further improve osteogenic performance by integrating pores at multiple length scales, thereby enhancing *in vivo* osseointegration. Current studies also suggest that effective coupling of micro- and macro-scale features is important for optimizing scaffold function. Accordingly, future work should clarify how microstructural design interacts with macroscopic mechanical properties to achieve improved biological integration.

Based on this PRISMA-ScR–guided synthesis of preclinical evidence, the field has identified design-relevant trends that may inform strategies for bone defect repair and support translational research in regenerative medicine. However, the current evidence base remains dominated by *in vitro* and animal studies conducted under controlled conditions. Further progress will require rigorous biomechanical validation, standardized experimental protocols and reporting, and ultimately well-designed clinical studies to establish safety and efficacy for specific indications.

## 10 Prospect

Titanium and its alloys are currently among the most widely used materials for orthopedic implants due to their excellent mechanical strength, natural corrosion resistance, and acceptable biocompatibility. Additive manufacturing (AM) meets the demands of complex implant structures, offering unprecedented opportunities for customized medical implants. Topological optimization has emerged as a powerful digital tool for optimizing structural and material designs. The integration of these techniques may enable the design and manufacture of implants with improved mechanical and osseointegration performance. However, several challenges remain in enhancing the performance of porous titanium alloy scaffolds:

- Multi-parameter Optimization: Future studies should move beyond single-factor analyses and adopt integrated design frameworks that account for interactions among pore geometry, porosity, strut diameter, and multiscale features. Data-driven and computational approaches may help identify robust design windows for specific anatomical sites and loading conditions.
- Structure–surface synergy and evaluation standardization: Biological outcomes are jointly governed by architecture and surface state. Improving the uniformity and long-term stability of surface functionalization within deep interconnected pores, together with standardized reporting of surface conditions and *in vivo* protocols, is essential for cross-study comparability.
- Mechanical reliability and long-term performance: Recent evidence has begun to quantify the fatigue behavior of additively manufactured Ti6Al4V struts/lattices across build orientations and mean-stress conditions, and has supported the development of image- and model-informed frameworks for fatigue-life prediction of lattice geometries (Murchio et al., 2024; Stammkötter et al., 2025; De Biasi et al., 2024). Key challenges remain, including ensuring fatigue resistance and damage tolerance in load-bearing applications, maintaining the stability of interfaces and surface modifications under physiological cyclic loading, and preserving sufficient permeability for tissue ingrowth. Future work should establish robust design–processing–performance correlations to improve long-term mechanical reliability under dynamic loading.
- Customizable stochastic and multiscale architectures: Stochastic and multiscale porous designs may better balance mechanical support and biological performance. Developing reproducible design rules and manufacturing strategies for patient-specific scaffolds remains an important direction.
- Translational pathways: Progress toward clinical adoption requires more physiologically relevant *in vitro* models, well-designed animal studies, and ultimately clinical trials with clearly defined indications and outcome measures. Close collaboration among engineers, biologists, and clinicians will be crucial for accelerating translation.

In summary, porous titanium alloy scaffolds are promising; however, future advances should prioritize standardization, robustness, and clinically relevant validation to bridge the gap between preclinical evidence and routine clinical use.
